# Supplementary material for: Cryo-EM structures of a pentameric ligand-gated ion channel in liposomes
Source: bioRxiv. 2025 Jun 2:2025.03.21.644626. Preprint. [Version 2] doi: 10.1101/2025.03.21.644626 (PMC12157481; doi:10.1101/2025.03.21.644626)
Supplement: Supplement 1 [file NIHPP2025.03.21.644626v2-supplement-1.pdf]

bioRxiv preprint doi: https://doi.org/10.1101/2025.03.21.644626; this version posted June 2, 2025. The copyright holder for this preprint (which was not certified by peer review) is the author/funder, who has granted bioRxiv a license to display the preprint in perpetuity. It is made available under aCC-BY 4.0 International license.

Supplementary Figure 1

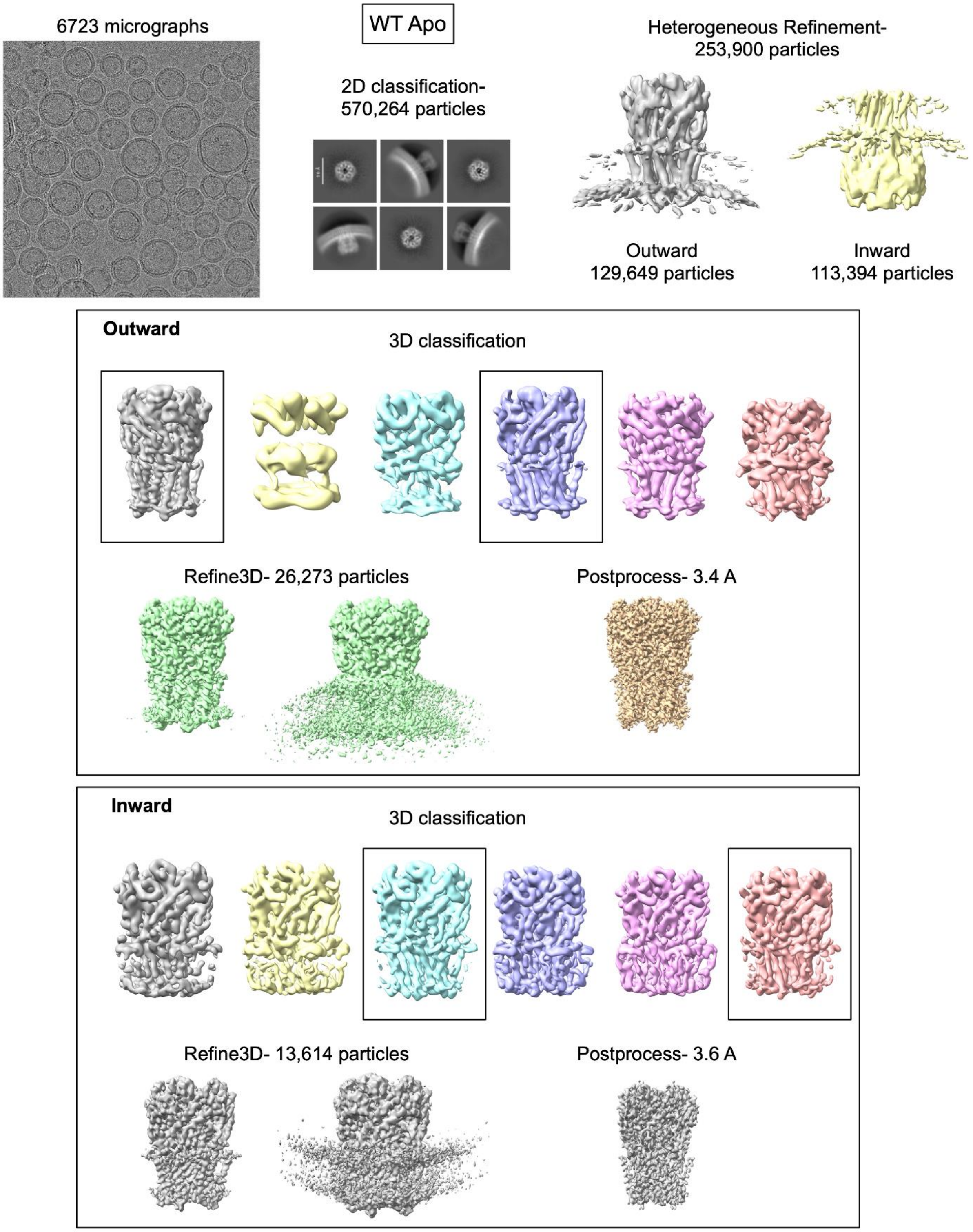

bioRxiv preprint doi: https://doi.org/10.1101/2025.03.21.644626; this version posted June 2, 2025. The copyright holder for this preprint (which was not certified by peer review) is the author/funder, who has granted bioRxiv a license to display the preprint in perpetuity. It is made available under aCC-BY 4.0 International license.

Supplementary Figure 1: Summary of single particle cryo-EM analysis of WT ELIC in liposomes. After multiple rounds of heterogeneous refinement in CryoSPARC to separate particles into inward- and outward-facing populations, multiple rounds of 3D classification were performed in Relion-5 to yield the final maps. The results from the final heterogeneous refinement and 3D classification jobs are shown.

bioRxiv preprint doi: https://doi.org/10.1101/2025.03.21.644626; this version posted June 2, 2025. The copyright holder for this preprint (which was not certified by peer review) is the author/funder, who has granted bioRxiv a license to display the preprint in perpetuity. It is made available under aCC-BY 4.0 International license.

Supplementary Figure 2

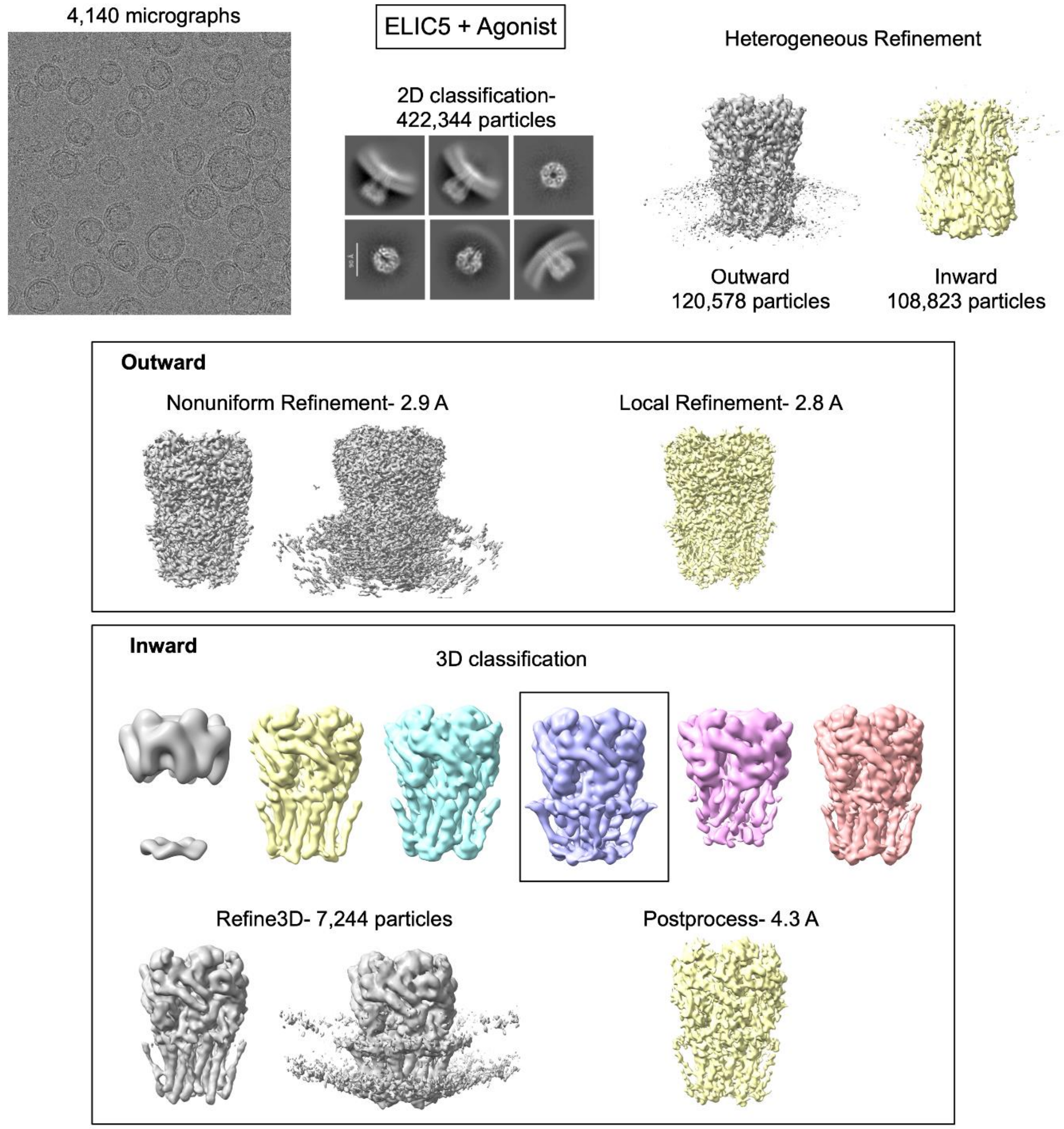

Supplementary Figure 2: Summary of single particle cryo-EM analysis of ELIC5 with agonist in liposomes, similar to Supplementary Figure 1.

bioRxiv preprint doi: https://doi.org/10.1101/2025.03.21.644626; this version posted June 2, 2025. The copyright holder for this preprint (which was not certified by peer review) is the author/funder, who has granted bioRxiv a license to display the preprint in perpetuity. It is made available under aCC-BY 4.0 International license.

Supplementary Figure 3

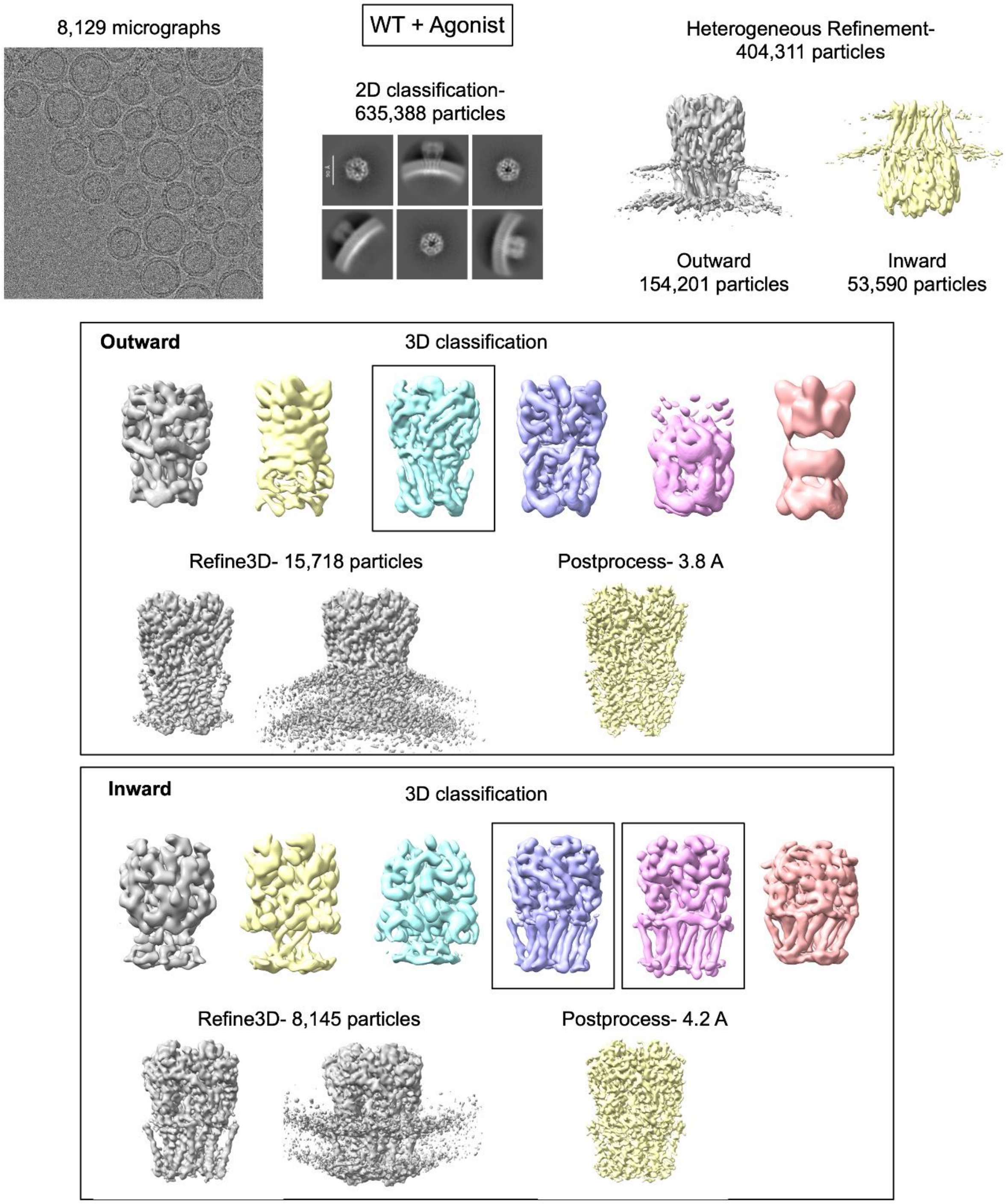

Supplementary Figure 3: Summary of single particle cryo-EM analysis of WT ELIC with agonist in liposomes, similar to Supplementary Figure 1.

bioRxiv preprint doi: https://doi.org/10.1101/2025.03.21.644626; this version posted June 2, 2025. The copyright holder for this preprint (which was not certified by peer review) is the author/funder, who has granted bioRxiv a license to display the preprint in perpetuity. It is made available under aCC-BY 4.0 International license.

Supplementary Figure 4

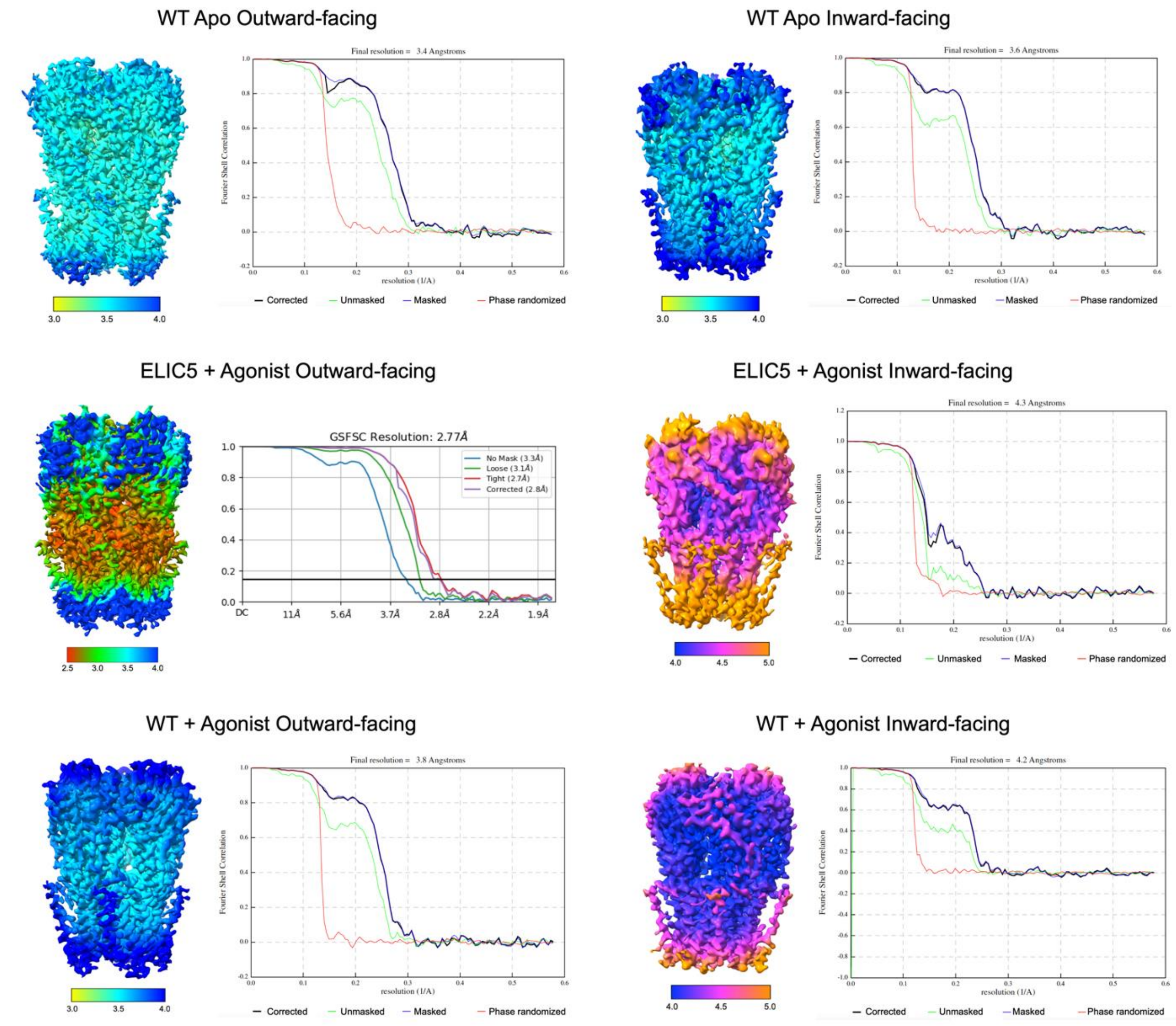

Supplementary Figure 4: Display of local resolution on sharpened maps and FSC curves from outward-facing and inward-facing ELIC structures in liposomes. The final map of outward-facing ELIC5 with agonist in liposomes was obtained from CryoSPARC. The remaining maps were obtained from Relion-5.

bioRxiv preprint doi: https://doi.org/10.1101/2025.03.21.644626; this version posted June 2, 2025. The copyright holder for this preprint (which was not certified by peer review) is the author/funder, who has granted bioRxiv a license to display the preprint in perpetuity. It is made available under aCC-BY 4.0 International license.

Supplementary Figure 5

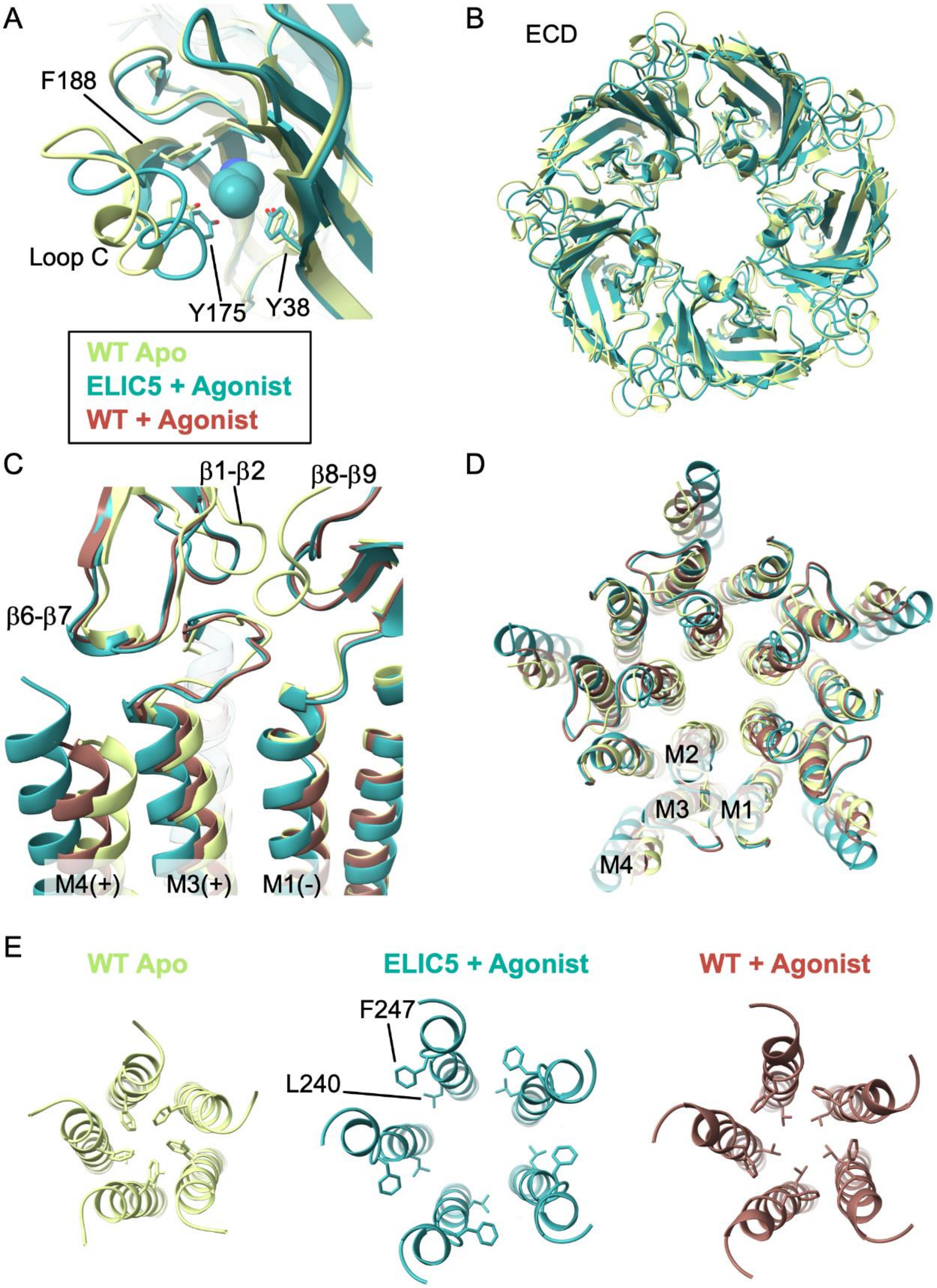

bioRxiv preprint doi: https://doi.org/10.1101/2025.03.21.644626; this version posted June 2, 2025. The copyright holder for this preprint (which was not certified by peer review) is the author/funder, who has granted bioRxiv a license to display the preprint in perpetuity. It is made available under aCC-BY 4.0 International license.

Supplementary Figure 5: Comparison of resting (WT apo), activated (ELIC5 + agonist) and desensitized (WT + agonist) structures of ELIC in liposomes. All images are from a global superposition of the structures. (A) View of the agonist/orthosteric binding site with bound propylamine. Loop C and three aromatic residues that interact with propylamine are labeled. The activated structure shows a contraction of the agonist binding site compared to the resting structure. (B) View of the extracellular domain (ECD) from the extracellular side along the pore axis. The activated structure shows a counter-clockwise twisting and slight contraction of the ECD. (C) View of the ECD-TMD interface. The β1-β2, β6-β7, and β8-β9 loops are labeled along with M4, and M3 and M1 from adjacent subunits. The ECD interfacial loops are similar between the activated and desensitized structures, but the transmembrane helices show a tilting and translation away from the pore axis that is greater in the activated compared to the desensitized structure. (D) View of the TMD from the extracellular side along the pore axis. (E) View of the pore-lining M2 showing the rotation of the F247 and L240 in the activated structure compared to the resting and desensitized structures.

bioRxiv preprint doi: https://doi.org/10.1101/2025.03.21.644626; this version posted June 2, 2025. The copyright holder for this preprint (which was not certified by peer review) is the author/funder, who has granted bioRxiv a license to display the preprint in perpetuity. It is made available under aCC-BY 4.0 International license.

Supplementary Figure 6

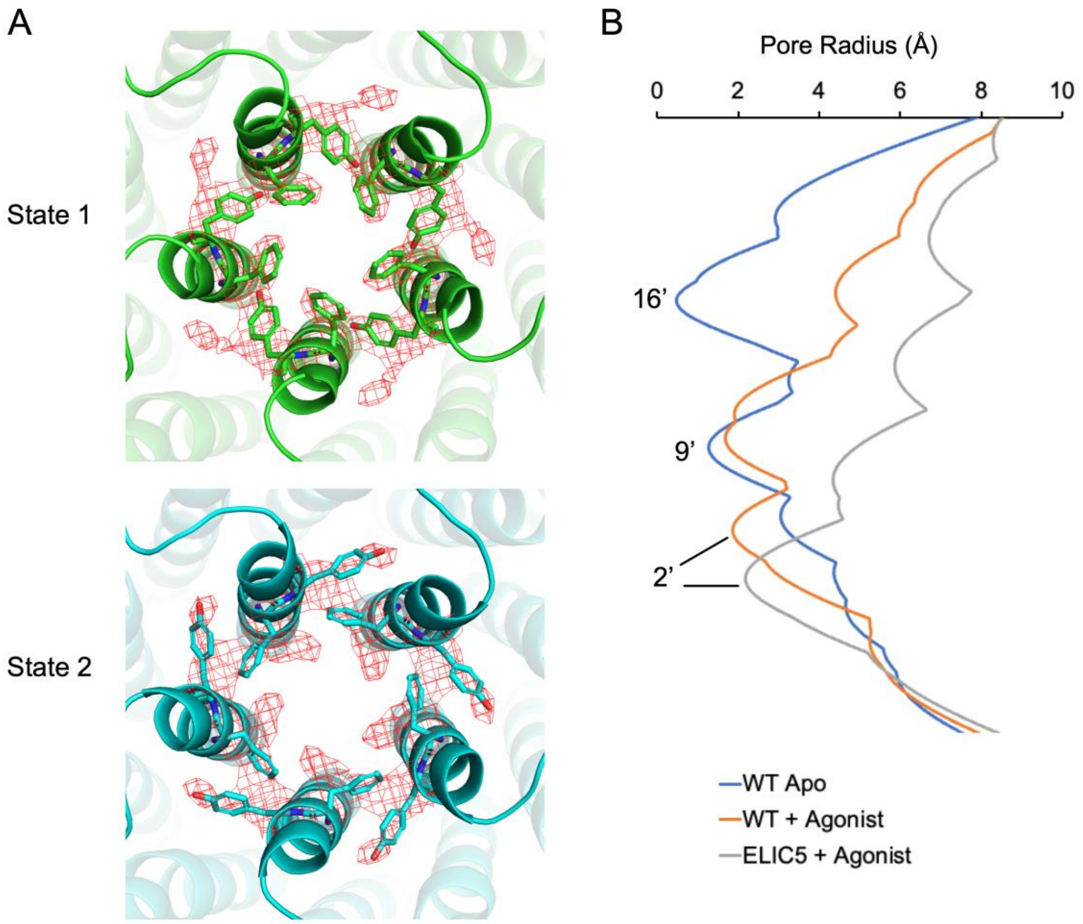

Supplementary Figure 6: (A) View of the cryo-EM map of WT ELIC with agonist in liposomes showing the fit for State 1 and State 2 models, which differ primarily in the side chain orientation of F247 and Y248. In State 1, F247 orients towards the pore axis, while in State 2, F247 orients towards the adjacent subunit. (B) Plot of the pore radius from the liposome structures (WT apo, WT with agonist state 2 and ELIC5 with agonist) generated using HOLE [45]. The pore-lining M2 residues at 16' (F247), 9' (L240) and 2' (Q233) are labeled.

bioRxiv preprint doi: https://doi.org/10.1101/2025.03.21.644626; this version posted June 2, 2025. The copyright holder for this preprint (which was not certified by peer review) is the author/funder, who has granted bioRxiv a license to display the preprint in perpetuity. It is made available under aCC-BY 4.0 International license.

Supplementary Figure 7

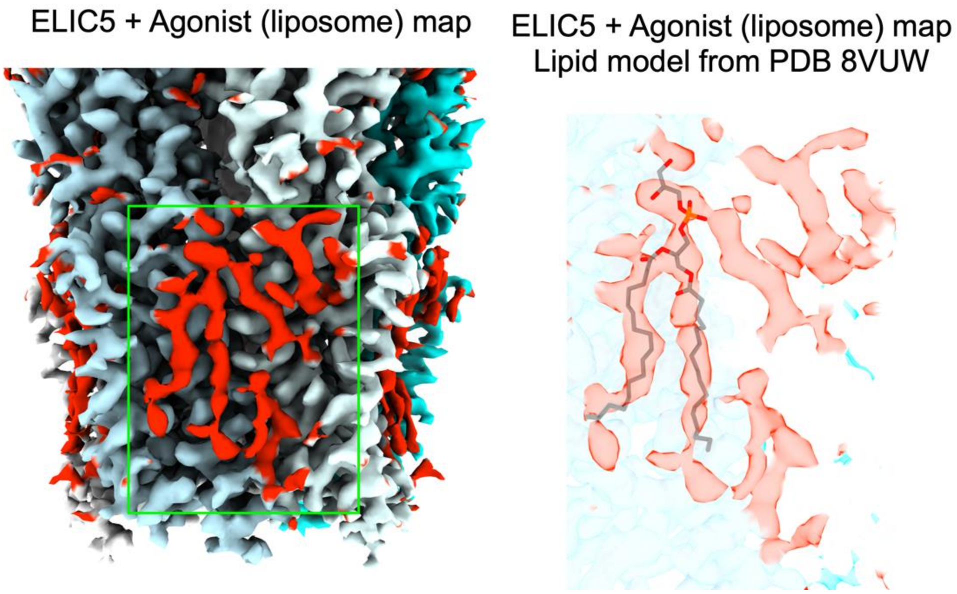

Supplementary Figure 7: Cryo-EM map of ELIC5 with agonist in liposomes with non-protein lipid-like densities colored in red. The lipid-like densities in the green box are shown on the right along with the phospholipid from PDB 8VUW (structure of agonist-bound ELIC5 in MSP1E3D1 nanodiscs). This image was produced by fitting ELIC5 from 8VUW in the ELIC5 map from liposomes, and displaying the phospholipid from 8VUW with the non-protein densities from the map of ELIC5 in liposomes.

bioRxiv preprint doi: https://doi.org/10.1101/2025.03.21.644626; this version posted June 2, 2025. The copyright holder for this preprint (which was not certified by peer review) is the author/funder, who has granted bioRxiv a license to display the preprint in perpetuity. It is made available under aCC-BY 4.0 International license.

Supplementary Figure 8

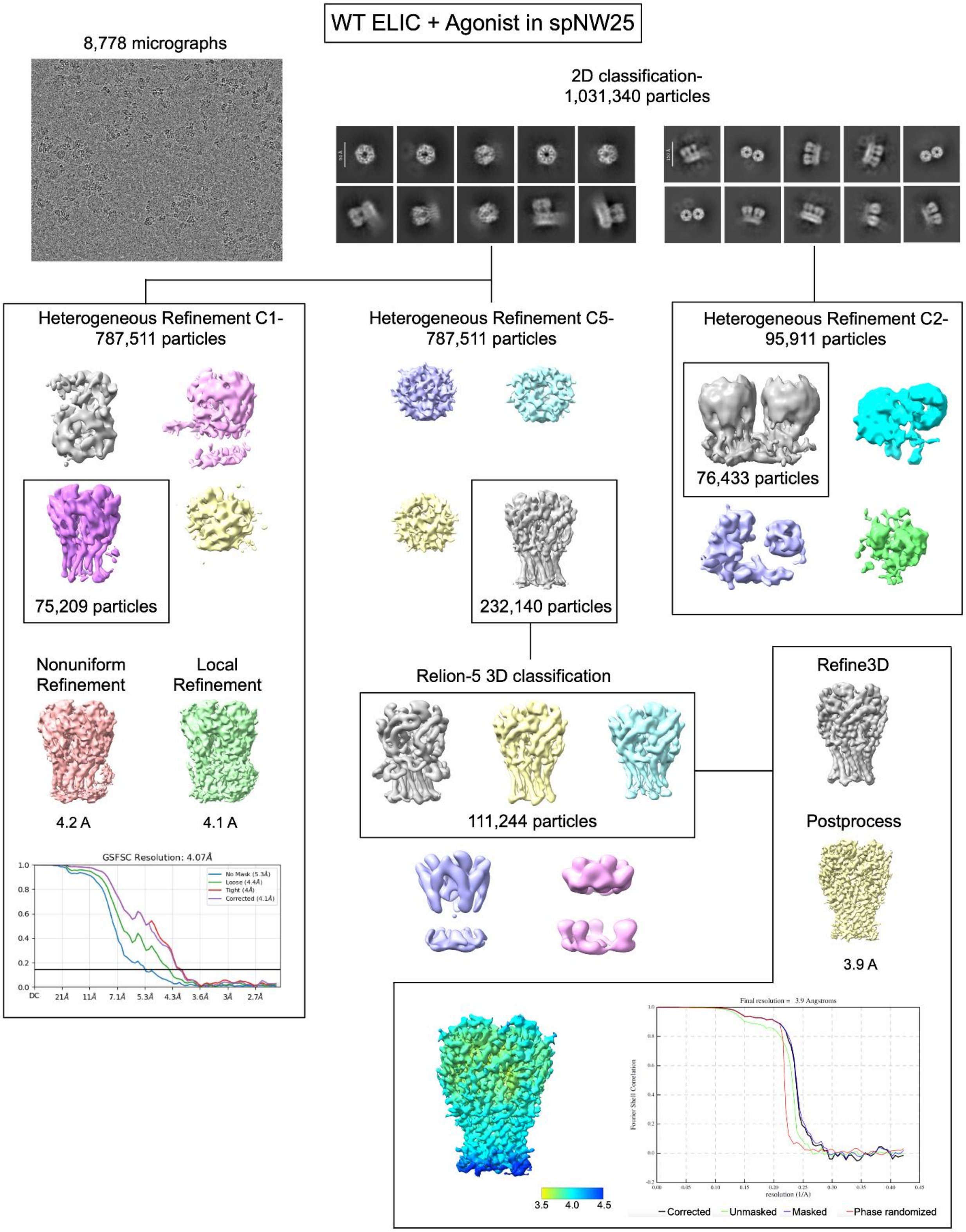

bioRxiv preprint doi: https://doi.org/10.1101/2025.03.21.644626; this version posted June 2, 2025. The copyright holder for this preprint (which was not certified by peer review) is the author/funder, who has granted bioRxiv a license to display the preprint in perpetuity. It is made available under aCC-BY 4.0 International license.

Supplementary Figure 8: Summary of single particle cryo-EM analysis of WT ELIC with agonist in spNW25 nanodiscs. 2D class averages were separated into single pentamers or dimers of pentamers. The particles consisting of single pentamers were processed with C1 symmetry in CryoSPARC only, or with C5 symmetry in CryoSPARC and Relion-5. Shown are the FSC curves for both C1 and C5 reconstructions, and the local resolution of the C5 map. The C1 map did not show significant asymmetry in the protein structure except for differences in the local resolution. Therefore, a model was only produced based on the C5 reconstruction, which had better resolution. The dimers of pentamers were separately processed in CryoSPARC with C2 symmetry, and this did not yield a high-resolution reconstruction.

bioRxiv preprint doi: https://doi.org/10.1101/2025.03.21.644626; this version posted June 2, 2025. The copyright holder for this preprint (which was not certified by peer review) is the author/funder, who has granted bioRxiv a license to display the preprint in perpetuity. It is made available under aCC-BY 4.0 International license.

Supplementary Table 1: Summary of cryo-EM data collection and refinement parameters

	ELIC state 1 with propylamine facing ECD outwards in liposomes with 2:1:1 POPC:POPE: POPG<br><br>EMD-49390<br><br>PDB 9NGQ	ELIC state 2 with propylamine facing ECD outwards in liposomes with 2:1:1 POPC:POPE: POPG<br><br>EMD-49391<br><br>PDB 9NGR	ELIC with propylamine facing ECD inwards in liposomes with 2:1:1 POPC:POPE: POPG<br><br>EMD-49392<br><br>PDB 9NGS	ELIC facing ECD outwards in liposomes with 2:1:1 POPC:POPE: POPG<br><br>EMD-49383<br><br>PDB 9NGF	ELIC facing ECD inwards in liposomes with 2:1:1 POPC:POPE: POPG<br><br>EMD-49384<br><br>PDB 9NGG
**Data collection and processing**					
Magnification	75000	75000	75000	75000	75000
Voltage (kV)	300	300	300	300	300
Electron exposure (e-/Å^2)	54.4	54.4	54.4	55.4	55.4
Defocus range (μm)	-1 to -2.4	-1 to -2.4	-1 to -2.4	-1 to -2.4	-1 to -2.4
Pixel size (Å)	0.865	0.865	0.865	0.868	0.868
Symmetry imposed	C5	C5	C5	C5	C5
Initial particle images (no)	635,388	635,388	635,388	570,264	570,264
Final particle images (no)	15,718	15,718	8,145	26,273	13,614
Map resolution (Å)	3.8	3.8	4.2	3.4	3.6
FSC threshold	0.143	0.143	0.143	0.143	0.143
**Refinement**					
Initial model used	PDB 8F34	PDB 8F34	PDB 8F34	PDB 8F35	PDB 8F35
Model resolution (Å)	3.8	3.8	4.2	3.5	3.7
FSC threshold	0.5	0.5	0.5	0.5	0.5

bioRxiv preprint doi: https://doi.org/10.1101/2025.03.21.644626; this version posted June 2, 2025. The copyright holder for this preprint (which was not certified by peer review) is the author/funder, who has granted bioRxiv a license to display the preprint in perpetuity. It is made available under aCC-BY 4.0 International license.

Map sharpening B factor (Å^2)	-130.301	-130.301	-138.788	-117.585	-130.345
**Model composition**					
Non-hydrogen atoms	12551	12551	12549	12585	12617
Protein Residues	1535	1535	1535	1535	1535
Ligands	5	5	5	0	0
**B factors (Å^2) (0.5)**					
Protein	13.55	13.55	29.12	27.02	25.93
Ligand	9.82	9.82	9.57	---------	---------
**R.m.s. deviations**					
Bond lengths (Å)	0.006	0.007	0.006	0.005	0.004
Bond angles (°)	1.099	1.132	1.167	1.083	1.012
**Validation**					
MolProbity Score	1.77	1.77	1.53	1.75	1.61
Clashscore	8.20	7.92	4	7	4.54
Poor rotamers (%)	0	0	0	0	0
**Ramachandran plot**					
Favored (%)	95.34	95.08	95.08	94.43	94.43
Allowed (%)	4.66	4.92	4.92	5.57	5.57
Disallowed (%)	0	0	0	0	0

bioRxiv preprint doi: https://doi.org/10.1101/2025.03.21.644626; this version posted June 2, 2025. The copyright holder for this preprint (which was not certified by peer review) is the author/funder, who has granted bioRxiv a license to display the preprint in perpetuity. It is made available under aCC-BY 4.0 International license.

	ELIC5 with propylamine facing ECD outwards in liposomes with 2:1:1 POPC:POPE:POPG<br><br>EMD-49382<br><br>PDB 9NGC	ELIC5 with propylamine facing ECD inwards in liposomes with 2:1:1 POPC:POPE:POPG<br><br>EMD-49385<br><br>PDB 9NGI	ELIC with propylamine in spNW25 nanodiscs with 2:1:1 POPC:POPE:POPG<br><br>EMD-49400<br><br>PDB 9NH4
**Data collection and processing**			
Magnification	75000	75000	120000
Voltage (kV)	300	300	200
Electron exposure (e-/Å^2)	56.9	56.9	46.6
Defocus range (μm)	-1 to -2.4	-1 to -2.4	-0.8 to -2.4
Pixel size (Å)	0.868	0.868	1.184
Symmetry imposed	C5	C5	C5
Initial particle images (no)	422,344	422,344	1,031,340
Final particle images (no)	120,578	7,244	111,244
Map resolution (Å)	2.8	4.3	3.9
FSC threshold	0.143	0.143	0.143
**Refinement**			
Initial model used	PDB 8VUW	PDB 8VUW	PDB 8F34
Model resolution (Å)	3.1	4.1	4.2
FSC threshold	0.5	0.5	0.5
Map sharpening B factor (Å^2)	-121.1	-156.539	-258.138
**Model composition**			

bioRxiv preprint doi: https://doi.org/10.1101/2025.03.21.644626; this version posted June 2, 2025. The copyright holder for this preprint (which was not certified by peer review) is the author/funder, who has granted bioRxiv a license to display the preprint in perpetuity. It is made available under aCC-BY 4.0 International license.

Non-hydrogen atoms	12722	12729	11320
Protein Residues	1550	1550	1375
Ligands	5	5	5
**B factors (Å^2) (0.5)**			
Protein	78.30	139.9	32.33
Ligand	59.82	93.71	16.82
**R.m.s. deviations**			
Bond lengths (Å)	0.005	0.006	0.006
Bond angles (°)	1.083	1.189	1.169
**Validation**			
MolProbity Score	1.22	1.94	2.29
Clashscore	2.33	11.19	20.80
Poor rotamers (%)	0	0	0
**Ramachandran plot**			
Favored (%)	96.75	94.48	92.31
Allowed (%)	3.25	5.52	7.69
Disallowed (%)	0	0	0

bioRxiv preprint doi: https://doi.org/10.1101/2025.03.21.644626; this version posted June 2, 2025. The copyright holder for this preprint (which was not certified by peer review) is the author/funder, who has granted bioRxiv a license to display the preprint in perpetuity. It is made available under aCC-BY 4.0 International license.

Supplementary Table 2:

	**M1 (W204 to L225)**	**M2 (F228 to T249)**	**M3 (V261 to F282)**
**WT ELIC with agonist in liposomes**	32.3 Å	32.5 Å	31.0 Å
**WT ELIC with agonist in spNW25**	30.5 Å	31.9 Å	30.2 Å
**WT ELIC with agonist in spMSP1D1 (PDB 8F34)**	32.0 Å	32.2 Å	30.6 Å

Supplementary Table 2: Distance along the pore axis (i.e. perpendicular to the lipid membrane) between the indicated residues (Cα atoms) for each structure. The measurements show changes in the height of each transmembrane helix along the pore axis.

bioRxiv preprint doi: https://doi.org/10.1101/2025.03.21.644626; this version posted June 2, 2025. The copyright holder for this preprint (which was not certified by peer review) is the author/funder, who has granted bioRxiv a license to display the preprint in perpetuity. It is made available under aCC-BY 4.0 International license.

Supplementary Table 3:

	WT ELIC with agonist		
	**MSP1E3D1 (PDB 8D66)**	**spMSP1D1 (PDB 8F34)**	**Liposome (PDB 9NGQ)**
**TMD Interface (area)**	1176.7 Å	1176.8 Å	1096.3 Å
**Buried surface area between M4 and the rest of ELIC**	771.7 Å	757.7 Å	732.7 Å
**Total buried surface area**	24864.7 Å	25183.4 Å	23947.3 Å
	**ELIC5 with agonist**		
	**MSP1E3D1 (PDB 8VUW)**	**spNW15 (PDB 8TWV)**	**Liposome (PDB 9NGC)**
**TMD Interface (area)**	1171.1 Å	1119.0 Å	1091.7 Å
**Buried surface area between M4 and the rest of ELIC**	842.0 Å	986.2 Å	870.3 Å
**Total buried surface area**	25312.0 Å	25065.1 Å	24166.5 Å

Supplementary Table 3: Measurements of the area at the interface between subunits in the TMD and the total buried surface area were determined using the PDBePISA server [46], and the buried surface area between M4 and the rest of the ELIC protein was determined using ChimeraX1.6.1.
